# Epithelial Dysplasia at Excision Margins of Oral Squamous Cell Carcinoma: A Review on Relationship to Clinicopathological Parameters and Prognosis

**DOI:** 10.31557/APJCP.2021.22.8.2313

**Published:** 2021-08

**Authors:** Nimna H. Senarath, Primali R Jayasooriya, Bogahawatte S.M.S. Siriwardena, Wanninayake M. Tilakaratne

**Affiliations:** 1 *Department of Community Dentistry, Faculty of Dental sciences, University of Peradeniya, Sri Lanka. *; 2 *Department of Oral Pathology, Faculty of Dental Sciences, University of Peradeniya, Sri Lanka.*; 3 *Department of Oral and Maxillofacial Clinical Sciences, Faculty of Dentistry, University of Malaya, Malaysia. *

**Keywords:** Epithelial dysplasia, oral squamous cell carcinoma, excision margin, prognosis

## Abstract

**Background:**

Epithelial dysplasia (ED) at oral cancer excision margins is a frequent finding. Dysplastic epithelium at excision margins may not be similar to dysplasia in Oral potentially malignant disorders (OPMD) as malignant transformation has already taken place. Therefore, management of ED at excision margins should be different to that of OPMD. ED creates a dilemma in relation to further management of cancer patients, since there are no accepted guidelines. Therefore, the objective of this review is to analyze existing literature and to arrive at evidence based recommendations for the management of ED at excision margins.

**Methods:**

A comprehensive string was run on PubMed, Medscape and Medline. The final outcome included 113 studies. Finally, the most relevant 10 articles were critically assessed for inclusion and exclusion criteria against various parameters.

**Results and Conclusions:**

Severe and Moderate ED need re-excision in order to improve prognosis. There is not enough sound evidence for the management of Mild ED at excision margins of oral squamous cell carcinoma. Guidelines for the management of ED at excision margins should be formulated after comprehensive multi center studies using lager cohorts of patients.

## Introduction

Oral squamous cell carcinoma (OSCC) is a common malignancy in some parts of the world which carries a high level of morbidity and mortality. Even though the treatment aspects have evolved over the past two decades, the 5-year survival rate is around 50% (Jemal et al., 2011) -60% (Siegel et al., 2013).

Epithelial dysplasia (ED) is identified as an indicator of increased probability of occurrence of OSCC, particularly in oral potentially malignant diseases (OPMD). Rate of transformation is stated as 10.3% (Mehanna et al., 2009) - 12 % (Raibel et al., 2017) within a mean duration of 4.3 years (Mehanna et al., 2009). However, it is also a frequent finding the surgical excision margins of already evolved OSCC. Surgical excision of an OSCC is carried out with the intent of complete excision of tumor with clear margins. The rationale of this guideline is to ensure recurrence free survival. There are number of clinical and histopathological factors identified as indicators of prognosis. Among these, ED at excision margins is also an important parameter, which requires further investigation.

Age, sex, risk habits and anatomical site of the OSCC are some clinical features associated with prognosis of OSCC and they may relate to ED at the margins as well .WHO defines tumour size, nodal status and distant metastasis as important prognosticators. Histological risk factors with such implications are; non-cohesive pattern of invasion, perineural and lymphovascular invasion, bone invasion, and depth of invasion. During excision of OSCC, there is a possibility of finding ED at the mucosal margins. As suggested in the given guidelines (Table 1), this can be incorporated into the histopathology report. Therein, it is also possible to include the grade of dysplasia as mild, moderate or severe, according to the WHO classification of oral epithelial dysplasia 2017. Newer studies on evaluation of influencing factors of the surgical margin indicate the necessity of considering presence and grade of ED in OSCC excision margins (He et al., 2017). Literature state that ED at margin as involved margins when the grade is ca in situ (Ravasz et al., 1990) while some exclude ED completely (Woolgar et al., 1999). There is no adequate literature to clinically guide surgeon regarding the management of excision margins, set in a status of field cancerization (Hinni et al., 2013). Nevertheless, since ED is considered as a vulnerable condition, capable of progressing into an OSCC in unhealthy environment, there are accepted guidelines for management of OPMDs with ED. In the binary system of classifying ED, low risk (mild/moderate ED) maybe managed by means of habit intervention and observations while high risk (severe ED) is subjected to excision (Raibel et al., 2017). Some moderate ED maybe considered as high risk as well, depending on the architectural and cytological features. The application of such a measure at excision margin requires further investigation. 

Adjuvant radiotherapy and chemotherapy are indicated commonly for patients with aggressive features such as invasive tumor at excision margin, positive cervical lymph nodes, extra-capsular spread of tumor in lymph nodes, following primary surgical excision. It may be a subjective decision for ED at margins.

Thus it is evident that management of ED in excision margin of OSCC is ambiguous. The objective of this review is to evaluate all existing and available literature to analyze common practices and their outcomes when ED is diagnosed at surgical margins and to clarify the controversies in the subject. Further, it will help in the development of evidence based guidelines in managing epithelial dysplasia at excision margins of OSCC. 

## Materials and Methods

This review is based on the available literature as at Dec, 2020. An initial search string was carried out in 2019 and 2020, for articles with the following keywords; [histolopathology]/ [histological], [prognostic indicators]/ [prognosis], with [dysplasia] in [resection margin], [excision margin], [surgical treatment], and [oral squamous cell carcinoma]. The following search engines and databases were used; Google scholar, Medscape, Researchgate, Hindawi and Pubmed. A total of 4 publications were presented directly with the keywords. These articles were obtained along with 6 more relevant articles, and the reference lists were scanned for related literature, manually by the authors. A total of 123 articles were listed using the terminology in their headings. Research articles that had not referred to the margin status as a prognostic indicator were excluded following the initial reading of their abstracts. A final collection of 81 articles were obtained in full text. There were 13 review/meta analyses with 68 original research papers. The articles with reference to excision margin, histopathological parameters, or margin width in their abstracts were scanned through to finalize their relevance for this review. The studies which aimed to assess margin width too were evaluated to clarify their definitions of the margin widths. Thereby the articles which have considered histologically diagnosed epithelial dysplasia at cancer excision margins were included in the review. Finally, a total of 10 articles which were directly related to the objectives and 10 other papers with some sections relevant to the area of investigation included in the analysis. Based on the available data and strength of evidence , a narrative type review was carried out.

## Results

Prevalence of ED at excision margins was observed in a wide range from 2.26% (Loree and Strong,1990) to 46.1% (Jerjes et al., 2010). This wide variation in prevalence is dependent on multiple factors. Some researches with extensive analysis on surgical excision margin status and its prognostic implications have not incorporated ED (Gensler et al., 2005; Smits et al., 2016) whilst some studies have included assessment of ED at the margin. However, it is not mentioned in results or in any other sections in the publication (Gensler et al., 2005). This emphasizes the fact that due attention has not been given to this important prognostic parameter in most related studies. All the studies were retrospective in nature and two studies have not stated details of study design (Kurita et al., 2010; Woolgar et al., 1995). There is a significant lack of prospective studies and it is a major drawback to arrive at clinically applicable conclusions. 

Categorization of ED varies in different studies. The WHO guidelines 2017 has modified the classification of ED including Ca in situ alongside severe ED, considering the management implications in patients with OPMD. The introduction of binary system, which categorizes ED in to low grade and high grade, may help clinicians’ decisions on management. However, new research is needed including the binary system of grading dysplasia, at the excision margins as currently there are no studies of such nature.

A survey carried out among members of the American head and neck society revealed some interesting facts about margin assessment in OSCC. Most indicated that ca in situ was a positive margin (83%) whereas a minority considered margin containing any grade of dysplasia (17%) as a positive margin (Larsen et al., 2009). It is important to note that studies 1, 2 and 3 (Table 2) excluded severe dysplasia and considered mild and moderate dysplasia as ED. Studies 4, 5 and 6 excluded ca in situ and separated mild, moderate and severe ED. Studies done by Montebugnoli et al., (2014) included all grades of dysplasia including ca in situ in epithelial precursor lesion category whereas Wong et al., (2012) separated patients with dysplasia/ ca in situ at the margin, from dysplasia associated with the tumor. Jerjes et al., (2010) used two categories, namely dysplasia at the margin (mild/moderate) and severe dysplasia. This arbitrary categorization leads us to lack of evidence based management.

Further, it is not uncommon to observe that even severe ED and Ca in situ at the margin of OSCC, patients were managed similar to OPMD patients, through re-excision or adjuvant therapy in many treatment centers. Severe ED in OPMD progresses into OSCC at a rate of 39% within a duration of 15 years, while the rates for mild and moderate ED are 6% and 18%, respectively (Raibel et al., 2017). It is important to emphasize the fact that ED at excision margins may not behave in a similar way to ED in OPMD as the former has already progressed into OSCC. Local recurrence rates with ED are reported as 17.6% (Chen et al., 2019), 69% (Jerjes et al., 2010) and 5% (Montebugnoli et al., 2014). This wide range may be related to the progression rates of different grades of ED. Mild ED has shown a LR of 35.71% (Gokaravapu et al., 2017) whilst it is 42.9% for severe ED. However, moderate ED has no recorded LR rate. Comparing to progression rates of OPMD, there are higher rates of transformation of ED at margins of OSCC patients. Therefore, it shows that ED at excision margins are more aggressive in terms of transformation and it is advisable to treat them more radically than the same grade of ED in OPMD.

*Survival and recurrence related aspects of ED at margins*

Different studies have used survival and recurrence data ranging from 1 to 5 years. This weakens the reliability of a comparative analysis. Chen et al., (2019) stated that there is a significant difference in DFS (Disease free survival), OS (Overall survival) and LC (Local control) among mild, moderate and severe ED. However, they also mentioned that presence of ED is not an independent risk factor for DFS (p=0.43) and OS (p=0.71). Considering the relationship between ED and recurrence, there is a serious lack of studies to determine the significance. The importance of all these evaluations should be to understand the significance of ED at margin to overall survival of OSCC patients. Chen et al., (2019) stated a significant association of overall survival to ED at margins when compared to patients with close margins. Gokaravapu et al., (2017) mentioned that moderate ED is significant for overall survival, after univariate and multivariate analyzes for different parameters. However, four other studies state that there is no such significance of ED at margins for overall survival of OSCC patients (Table 2). 

DFS was significantly associated with ED at margin in one research (54.7% p<0.001) (Chen et al., 2019), while it was not significant in another 41.37% (p=0.5)(Wong et al.,2012). There is not enough sound evidence to draw firm conclusions.

**Table 1:** Classification of OSCC Excision Margins

Guidelines	Clear	Close	Involved /positive	Other
American college of pathologists (Raja et al., 2017)	Commonly used cut off points to define close margins are 5 mm in general and 2 mm with respect to glottic larynx. However, values ranging from 3 mm to 7 mm have been used with success and for glottic tumors as low as 1 mm. Distance of tumour from the nearest margin should be recorded.			Mild dysplasia at a margin is considered low risk and negative, while severe dysplasia at margin is considered high risk and positive. Moderate dysplasia at margin is implies an intermediate risk and is reported as positive
Royal college of pathologists, UK (Helliwell and Woolgar,2013)	>5mm from the tumor	1-5mmfrom the tumor	<1mm from the tumor	Additional category –Epithelial precursor lesion: included severe ED & Ca in situ as well. Excluded patients with invasive Ca within 5mm.
National comprehensive cancer network guidelines (2017)			Ca in situ or invasive ca at the margin	
Batsakis (1999)and Sutton (2003).	No evidence of tumor within 5mm	Tumor within 5mm but not at the margin	Frank tumor at the margin	

**Table 2:** Summary of Literature Review

Study	Total Number of Patients (n)	Patients with Epithelial dysplasia(PED)	Local control local recurrence (LR)/regional (RR)-(PED), Distant metastasis(DM), Second primary tumor(SPT)	Disease free survival of PED (DFS)	Overall survival: PED
1.Cheng et al., 2019 (Taiwan)**	1642	170 (10.35%)	LR : 30(17.65%)p<0.001 Between ED and positive margins.	At 5 years: 54.7% P<0.001	72% P<0.001
Follow up period: 5 yrs			RR: 20(11.76%)p=0.49	Between ED margin and positive margins,	Significant between ED margins and close margins.
			DM: 7(4.12%)p=0.01	not clear or close margins	Not with clear margins
			SPT: 21(12.35%)p=0.14	5 years :	
				DFS and OS : not significantly different in patients with dysplastic and clear margins. (p==0.37 and p= 0.38)	
2.Gokaravapu et al., 2017 (India)	425	57(13.41%) Mild ED 28	Loco-regional recurrence : 16/102(not significant)	Mild/moderate/no ED association with survival p=0.06 Mild+moderate ED vs no ED for survival : p=0.043	
Follow up period: 33-69 months		moderate ED : 29		Moderate ED was significant: p<0.05 in multivariate and univariate analysis.	
3.Weijers et al., 2002. (Netherlands) Follow up period: 5 yrs	37 Tongue & Floor of the mouth	7 (18.9%)	LR: 5/7 (p<0.01) <7% at 5 year follow up.		
4.Sopka et al., 2013. (USA)*	126	48(37%)	At 5years	At 5yrs	
Follow up 1-250 months	All OSCC tongue		LC: 80%vs 60(ED)% p=0.12 DFS: 78% vs60% (ED) p=0.17.	77%	
5.Kurita et al., 2010. (Japan)**	148	13(8.8%)	At 5 yrs		
			LC: 81.8%		
Follow up:5 yrs			P<0.001		
6.Pu et al.,2016(China)*	539	108(20%)	Have analyzed in different grade: see below		
Follow up :150 months					
7.Montebugnoli et al., 2014. (Italy)	180	21 (11.6%)	LR of EPL:		
Minimum follow up: 12 months		Epithelial precursor lesions*(EPL)	5%(p=9.204 SPT in EPL: 43%(p=0.003)		
8.Jerjes et al., 2010. (UK)	115	ED:53(46.1%)	Death from LR and DM: significant association with ED p=0.005		
Follow up :3 and 5 years			Recurrence with ED: 30(69.8%) p<0.001		
			DM with ED: 9(17%)		
			Regional metastasis: 8(15.1%0		
9.Wong et al., 2012. (UK)	192	All ED	Ca in situ	Ca in situ	
Follow up: minimum 24months.		:82	:8/17 ,p=0.07	: 8/29,p=0.83	
		-42.70%	ED : 8/17 ,p=0.89	ED: 12/29,p=0.5	
10.Loree and Strong ,1990. (USA)	398	9(2.26%)	LR: 33%		

*, Frozen section analysis on all patients; **, Frozen section analyzed in some patients and not categorized accordingly

**Table 3:** Epithelial Dysplasia at Excision Margin

	Study	Patients	Prognosis	Remarks
Mild epithelial dysplasia	Chen et al.,2019.	53(31.18%)	-	No separate analysis on the effect of mild ED.Margin width not included along with grade of ED.
	Gokaravapu et al., 2017.	28/7(49.1)	Loco-regional recurrence: 10(p=0.307) Death :16(p=0.061)	Comprehensive analysis of mild ED against age, sex, tobacco use, site , OSCC differentiation , width, LVI,PNI, T stage, Neck status and Loco regional recurrence.
	Sopka et al., 2013.	15(31%)	At 5 years LC: 81% , DFS:81%, OS : 76%	Assessed for the impact on local control and disease free survival.
	Kurita et al., 2010.	5/13(38.5%)	No recurrence	Assessed only for local recurrence rate.
	Pu et al., 2016.	67(12.4%)	5yr : OS: 70.4%, RFS: 74.9%	Compared with negative margins- mild ED, with re excision was not predictive of a worse DFS p=0.959, mild ED without re excision was predictive of worse DFS p=0.014, and RFS p=0.010.
		21/67-40.3%) were re-resected	DFS:66.5%	
			Mild ED with re-excision vs mild ED: OS: 95.2% vs 50.3%, p<0.0001	
			DFS: 90.5% vs59.4% , p<0.0001	
			RFS: 100%vs 59.6%, p<0.0001	
Moderate epithelial dysplasia	Chen et al.,2019.	117(68.82%)	-	No separate analysis on the effect of moderate ED and margin width not included along with grade of ED.
	Gokaravapu et al., 2017.	29/57(50.8%)	Loco-regional recurrence: 6 Death: 23	Comprehensive analysis of moderate ED against age, sex, tobacco use, site, OSCC differentiation, width, LVI, PNI, T stage, Neck status and Loco regional recurrence.
	Sopka et al., 2013.	21(44)	At5years LC:49% p=0.02 DFS=49%, OS: 77%	Significance changed when moderate grouped together with severe. See below. Separate multivariate analysis: moderate ED significant predictor of LC p=0.03 and DFS p=0.036
	Kurita et al., 2010.	1/13(7.6%)	No LR	Assessed only for local recurrence rate.
	Y Pu et al., 2016.	23(4.3%)	At 5 years; OS: 86.1%, RFS: 77.3% ,DFS: 67.6%	Moderate ED against negative margins was predictive of worse RFS and DFS.
Severe epithelial dysplasia/ Carcinoma in situ	Chen et al., 2019.	41(24.26%)		Excluded
	Sopka et al., 2013.	12(25%)	LC: 54% DFS: 54% OS: 74%	Significance of severe ED only approached significance at 5 years, p=0.1, together with moderate ED it was a significant factor affecting LC p=0.02. Multivariate analysis: moderate and severe ED significant for loco-regional recurrence. P=0.009 and DFS p=0.008.
	Kurita et al., 2010.	7	LR: 42.9%	Assessed only for local recurrence rate.
	Pu et al., 2016.	18(3.3%)	OS:50% RFS: 34.5% DFS: 32.3%	Severe ED vs Negative margins was predictive of worse RFS and DFS.
	Jerjes et al.,2010.	72 severe ED	LR-	Dysplasia at margin is an excellent predictor of tumor spread.
			37/43 patients with severe ED.	
			Death from LR spread: 7/10(70%)	
			Death from DM:	
			10/11(90%)p=0.271	

**Figure 1:**
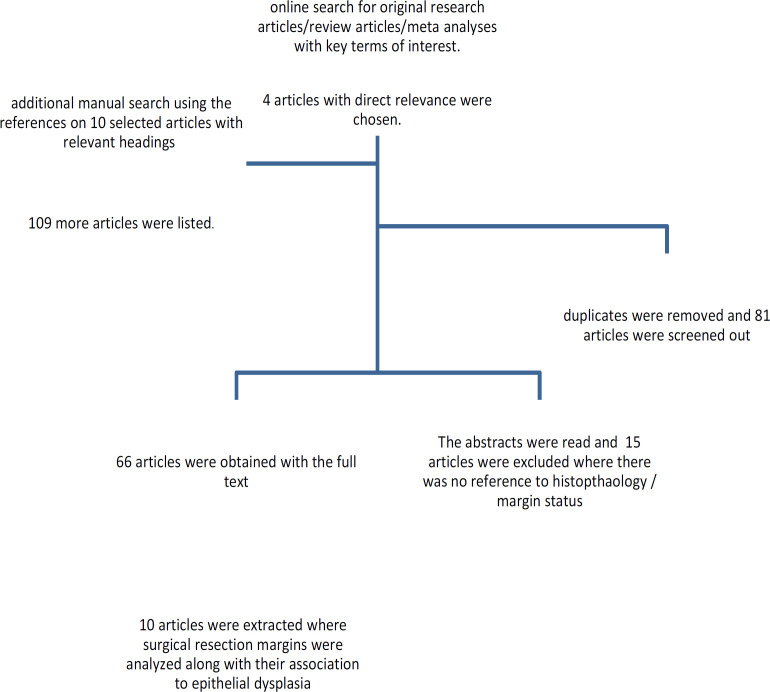
Methodology

## Discussion

Local recurrence is observed to be affected by ED in four studies whist others have stated ED as an independent risk factor. On the contrary, four other studies have not found a positive relationship(Table 2). Ca in situ was identified to have a significant relationship to recurrence. However, it can be accepted that compared to normal mucosa it is a risk to have ED at excision margin. But there is not enough evidence in the literature to quantify this risk. Upile et al., (2012) assessed the deleterious nature of invasive front and dysplasia at margin based on Byrne’s classification where they have observed 63/282 had severe dysplasia, while other grades were not specified and that dysplasia at excision margin was significantly associated with local recurrence (p<0.05, Hazard ratio: 0.418). Regional recurrence and Loco-regional control were devoid of any significant relationship to ED at excision margin (Chenetal., 2019) (Gokaravapu et al., 2017). However, according to others the relative risk of recurrence is five times higher for patients with dysplasia at margins when compared to patient devoid of dysplasia (Kurita et al., 2010). Therefore, tumour free and dysplasia free margins are the new heights in complete excision (Siegel et al., 2013). It can be assumed that regional recurrence may be more related to field cancerization rather than the margin status itself. Chen et al., (2019) and Jerjes et al., (2010) stated that ED at margin indicates poor prognosis and death from loco- regional and distant metastasis and concluded that ED at margin is an excellent predictor of tumor spread. 

Chen et al., (2019) stated that there is no significant association between second primary tumour and presence of ED at the margin of primary tumour. However, Montebugnoli et al., (2014) claimed there is a relationship between the two parameters. However, the latter had considered all ED grades collectively in one category, as epithelial precursor lesions, which included ca in situ as well. Such differences in classification render the results incomparable.

*Mild ED at margins*

One study categorized patients into two groups. Mild dysplasia which had been re-excised and mild dysplasia kept under observation (Table 3). A significant difference was observed in 5 year survival rate, DFS and recurrence free survival (RFS). In this sample, 21 patients with mild dysplasia had undergone re-excision while 46 patients had not. 

In studies with frozen section analysis, DFS was less in patients with ED at margins when compared to patients without ED, except for patients with mild ED (78% vs 81%) (Sopka et al., 2013). Mild dysplasia in the mucosal margin of a surgically removed tumour is weighed in a similar scale in the eye of a histopathologist who identifies it in an OPMD. Thus, most centers do not recommend additional intervention other than habit cessation. However, in this review several interesting revelations were noticed. An extensive research that included re -excision of mild ED in excision margin of some patients and comparing them to the test group with mild ED, demonstrated that it could benefit to excise mild ED as it improves RFS, DFS, and OS. Further, it is not clear whether these two groups were randomized in relation to other characteristics that can affect prognosis and it is also interesting to notice that when compared to negative margins, mild ED with re-excision had not shown a worse outcome. According to these findings one can argue on the importance of considering the presence of ED at margins when treating oral cancer. Yet, it is mentioned that mild dysplasia was not predictive of DFS or RFS when compared to other parameters at the margin (Pu et al., 2016). They further suggest that additional attention be drawn to dysplasia at the initial margin in OSCC including mild ED, and extended excision is suggested. Thus, it can be suggested that mild ED maybe be re-excised when feasible.

Mild or moderate dysplasia in mucosal margins relates to an over 50% chance of local recurrence within the first 5 years after excision of the primary tumor (Weijers et al., 2002). Sopka et al., (2013) stated that it is justifiable to attempt clearance of moderate dysplasia by additional excision at margin, despite added morbidity where possible reversal through postoperative adjuvant therapy may seem inadequate (Table 3). Moderate ED is identified to have a significance in predicting LC and DFS. Although moderate ED was defined as 2/3 involvement of the epithelium, in the WHO classification, it is a diagnosis with high possibility of variability (Senarath et al., 2019). Application of binary system may lessen the confounding nature and lead to a more reliable outcome. Overall, the available literature suggests that treating moderate ED at margins lead to better prognosis (Table 3).

*Severe ED at margins*

Amaral et al., (2004) in their study concluded that severe dysplasia is advised to be re excised at all times. It is known to have a significant increase in recurrence (Kurita et al., 2010). Studies showed that overall survival with severe dysplasia or positive margins result in worse outcome when compared to the group with negative margins for tumour (McMahon et al., 2003). One study stated that severe ED may not act as a significant prognosticator, when it is considered alone (Kurita et al., 2010). However, when it was grouped together with moderate ED, it was significant. It is important to note that both these studies excluded ca in situ. Some studies have excluded ca in situ and severe ED, and included only mild and moderate ED. Given the risk annotated to severe ED, it is acceptable to presume that higher the grade of ED, greater the possibility of transforming into an OSCC. These studies suggest that severe ED at margins need further treatment in order to achieve better prognosis (Table 3).

*Relationship between age and gender with ED at margins*

Categorization of patients with ED according to the age was observed only in two studies, while gender was assessed in three. Both had below and above 50 year age categories where they were unable to find a significant association. Two studies revealed that there is no significant association between occurrence of ED at excision margin to male or female gender (p=0.196) (Gokaravapu et al., 2017). However, Chen et al., (2019) stated otherwise. They identified a significant relationship between male gender and ED at margins 147(86.47%) vs. 23(13.53%) p=0.01. Due to the higher prevalence of habits and OSCC in males, it may be predictable that more male patients would present with ED at margins after surgery. while other studies agreed on the contrary (Gokaravapu et al.,2017). With the available evidence, it is not clear whether ED at margins has a difference in males and females. Further, it is not possible to predict the behavior of ED at margins according to gender with the available literature.

*Relationship between site of cancer and ED at margins*

Many investigators have not assessed ED at margins with reference to tumor site. However, it may be influenced by anatomical restrictions to surgical treatment. Weijers et al., (2002) assessed margins with ED between tongue (5 vs. 14) and floor of the mouth (2 vs. 16) and revealed that there was no significant relationship. Similarly, Gokaravapu (2017) showed that there was no statistically significant (p=0.801) relationship between different sites (Buccal mucosa, gingiva and tongue) and dysplasia at the margin. Woolgar et al., (1995) observed that there were no significant differences in the frequency of ED at margins, in relation to tumor site. Overall, literature supports that there is supportive evidence to accept that behavior of dysplasia at excision margins may not be significantly affected by the site of the primary OSCC. It is imperative to notice that OPMD in different sites are observed to behave differently which affirms the difference of ED in OPMD to ED at margins.

*Relationship between habits and ED at margins*

Even though habits are included in most of the studies, they have not considered ED to determine its relationship or impact on prognosis except for the study by Gokaravapu et al., (2017). It depicted that significantly large number of patients with mild (23/28) or moderate (13/29) ED at the margin were tobacco users The relationship of tobacco use and overall survival was not evident in this study since patients were not grouped according to past habits or current or continuous habits. However, important clinical relationship was present between moderate ED at margin with tobacco usage. Studies have not adequately assessed risk habits in terms of duration, type and cessation in patients with ED at margins to provide a clear opinion. However, it is reasonable to presume that patients with longer duration of chewing habit to have more field cancerization effect and ED.

*Relationship between size of the tumour and ED at margins*

Weijers et al., (2002) stated that there is no correlation between size of the tumor to ED at the margin. Grade of ED was considered only in one study where the association of T stage to ED was not statistically significant. A significant association was found between ED (mild and moderate) at margins to T1-T2 OSCC than T3-T4. However, Chen et al., (2019) and Gokaravapu et al., (2017) stated that there is no apparent relationship. 

Gokaravapu et al., (2017) observed that mild and moderate ED were mostly present in tumors with depth of invasion (DOI) of 3-9mm and more than 9 mm, but less in 1-3mm (p=0.27) category. Chen et al., (2019) observed that ED was more common in tumors with thickness more than 10mm (p<0.001). It could be possible to assume that thicker tumours require extended excisions in order to have dysplasia free margins. The surgeons may take precautions to avoid larger amounts of tissue loss for functional aspects and quality of life that may unintentionally limit the width of excision resulting in failure to obtain clear margins of tumour without ED. It could also be related to field cancerization in patients with prolong risk habits. However, available literature is inadequate to draw firm conclusions on the relationship between DOI and presence of ED at margins leading to management decisions. Size and thickness of the tumor apparently have inverse relationship to ED in margins which needs further clarification .

*Relationship between margin width, lympho-vascular spread, Perineural invasion and Neck status with ED at margins*

Margin width is a controversial parameter which is continuously probed in number of studies. Chen et al., (2019) categorized dysplastic margin (mild and moderate) against >5mm, <5mm and <1mm. ED was present in >5mm (75 : 44.12%) and <5mm (95: 55.88%) groups, which was not statistically significant. Similarly, Gokaravapu et al., (2017) categorized margin status as and concluded that patients with mild or moderate ED at the margin often had 1-3mm (p=0.005) margin width. Only a few studies included dysplasia in relation to margin width. Jones et al., (1992) included ED at the margin and tumor away from 2 mm, both as clear margins and concluded that positive margins were the ones significantly associated with recurrence. Therefore, it should be emphasized that a standard is necessary when incorporating margin width and ED in research. 

There were two studies which have assessed ED at margin with Lymphovascular invasion (LVI), Perineural invasion (PNI) and status of neck nodes. Chen et al., (2019) stated that compared with OSCC with LVI, OR for the presence of dysplastic margins in OSCC without LVI was, 1.51 (p=0.10). Gokaravapu et al., (2017) observed that there is no LVI in their patients with ED at margins. Chen et al., (2019) report that compared with patients with PNI, OR of patients with ED and without PNI was 1.48 (p=0.009). A similar observation was made by Gokaravapu et al., (2017), however it was not statistically significant. Further, No/N1 nodal status were more prevalent in patients with ED at margins compared to N2/N3 (p<0.001) (Chen et al., 2019). This suggests that OSCCs at lower stages may undergo less radical excision.

*Adjuvant therapy and ED at margins*

Chemotherapy/radiotherapy in patients with ED was evaluated in two studies included in this review. Chen et al., (2019) revealed a critical association between patients with ED and their response to RT. It was stated that there was a significantly worse outcome in this category of patients. (p=0.003). Kurita et al., (2010) also observed that 30% of the patients with ED who were subjected to RT, presented with local recurrence. Hinni et al., (2013) mentioned in their review, that adjuvant therapy improves loco regional control and improved overall survival in high risk patients with ED at margins Thus, it is necessary to rethink the response of ED in OSCC excision margin to RT in relation to prognosis. However, it seems necessary to evaluate low risk and high risk ED and their response to RT, for further understanding. It is also important to note that many studies defined their study population excluding all patients who underwent RT and CT prior to surgery or postoperatively. This limits the observations made in the actual environment.

*Strength of evidence*

The final articles that were analyzed in this review were ten observational cohort studies of retrospective nature. Assessment of the these articles on prognosis or risk were as follows ; moderately low risk -06, moderately high riskc-01 , high risk-03. Overall strength determination of overall strength of evidence: Moderate confidence. 

*Limitations of the existing literature*

Most studies are retrospective in nature, have less sample size and lack well-defined study design. There is a need to evaluate ED related to other clinical, histological and prognostic factors, rather than as an independent risk factor in order to formulate comprehensive treatment guidelines. 

There is a definite need for further investigation on classification of margin status (clear/close/involved) in relation to presence of ED. The available limited literature suggests that further treatment of excision margins with moderate and severe ED, lead to better prognosis. The studies are not conclusive for the same with regards to mild ED at margins. Furthermore, ED at margins requires prospective evaluation with the use of binary grading system to formulate evidence based guidelines as it may reduce the problems that exist in the present three tier grading system. Currently, there is no sound scientific evidence to suggest that ED at excision margins of an OSCC behaves in the same manner as ED in OPMD. Further, it can be stated that presence of ED at excision margins, contradicts the use of radiotherapy but it does not significantly influence the loco-regional recurrences.

## Author Contribution Statement

Study conception and design: WM Tilakaratne: Data collection, analysis and interpretation of results, and manuscript preparation: NH Senarath. Revised it critically for important intellectual content and editing: PR Jayasooriya, BSMS Siriwardena.

## Data Availability

Raw data can be produced upon request.

## Final manuscript assessment, and approved the version to be published

All authors.
